# Resistance to PARP-Inhibitors in Cancer Therapy

**DOI:** 10.3389/fphar.2013.00018

**Published:** 2013-02-27

**Authors:** Alicia Montoni, Mihaela Robu, Émilie Pouliot, Girish M. Shah

**Affiliations:** ^1^Laboratory for Skin Cancer Research, (CHU-Q) Hospital Research Centre of Laval University, Laval UniversityQuébec, QC, Canada

**Keywords:** poly(ADP-ribose) polymerase, PARP-inhibitors, synthetic lethality, potentiation of anti-cancer therapy, resistance to PARP-inhibitors, DNA damage, DNA repair

## Abstract

The pharmacological inhibitors of poly(ADP-ribose) polymerase (PARP) family of proteins have shown promising results in preclinical studies and clinical trials as a monotherapy or in combination therapy for some cancers. Thus, usage of PARP-inhibitors (PARPi) in cancer therapy is bound to increase with time, but resistance of cancer cells to PARPi is also beginning to be observed. Here we review different known and potential mechanisms by which: (i) PARPi kill cancer cells; and (ii) cancer cells develop resistance to PARPi. Understanding the lethality caused by PARPi and the countermeasures deployed by cancers cells to survive PARPi will help us rationalize the use of this new class of drugs in cancer therapy.

Recent clinical trials with poly(ADP-ribose) polymerase-inhibitors as a monotherapy or in combination therapy have shown promising results against different cancers (Lord and Ashworth, 2012). Therefore, their use in cancer therapy is likely to increase, resulting in the inevitable appearance of PARPi-resistant cancers (Chiarugi, 2012). Here, we first discuss different mechanisms by which PARPi can kill cancer cells and then review several known and potential mechanisms by which cancers can become resistant to PARPi.

## Mechanisms of Action of PARPi in Cancer Therapy

### PARP-1 as the principle target for therapeutic activity of PARPi

There are 18 members of the PARP family of proteins, but therapeutic effect of PARPi on cancer cells is observed only in conjunction with DNA damage; hence DNA damage-responsive PARPs are the most likely mediators of PARPi effect. Among three such PARPs, PARP-1 is the principle responder to DNA damage, as it rapidly reaches the damaged site and mounts a robust catalytic activation response that influences different cellular responses to DNA damage (Javle and Curtin, 2011; Yélamos et al., 2011; Gibson and Kraus, 2012). The activated PARP-1 splits the substrate nicotinamide adenine dinucleotide (NAD^+^) to release ADP-ribose, nicotinamide, and protons (Affar et al., 2002; Shah et al., 2011). PARP-1 then forms polymers of ADP-ribose (PAR) that post-translationally modify (i.e., PARylate) PARP-1 itself and selected target proteins to control a wide array of cellular processes, such as cell death, transcription, cell division, and DNA repair (Krishnakumar and Kraus, 2010). Among the DNA repair pathways, PARP-1 is widely recognized for its impact on the base excision repair (BER) and single strand break (SSB) repair pathways, but it also influences homologous recombination (HR) and non-homologous end-joining (NHEJ) repair of double strand breaks (DSB; Yélamos et al., 2011; De Vos et al., 2012). In addition, it also plays a role in mismatch repair (Liu et al., 2012) and more recently the nucleotide excision repair pathways (King et al., 2012; Luijsterburg et al., 2012; Pines et al., 2012; Robu et al., 2013).

In contrast to PARP-1, the other two DNA damage-responsive PARPs play a limited role in DNA damage responses. For example, PARP-2, in conjunction with PARP-1, has been shown to affect BER (Schreiber et al., 2002) and restart the stalled replication forks (Bryant et al., 2009). PARP-3 plays a role in NHEJ pathway in conjunction with APLF (Rulten et al., 2011) or PARP-1 (Boehler et al., 2011) and helps activation of PARP-1 (Loseva et al., 2010). In the context of the role of PARPi in inhibiting PARylation activity of PARPs, it is pertinent to note that PARP-2 has a very weak PARylation activity as compared to PARP-1, and many functions of PARP-2 and 3 are associated with PARP-1. Therefore, one could argue that the main target for PARPi is on the role of PARP-1 in DNA repair with possibly some effect on the roles of PARP-2 and 3. Finally, we should not exclude the possibility that the roles of PARP-1 in cell death and transcription are also involved in the therapeutic effect of PARPi.

### Competitive PARPi have consistent therapeutic activity

Most consistent results in clinical trials have been obtained with competitive PARPi, which are analogs of nicotinamide that compete with the substrate NAD^+^ to bind to the enzyme. Unlike weak inhibitory activity of nicotinamide, its derivatives ranging from the first generation 3-aminobenzamide to the third generation Olaparib and Rucaparib are better inhibitors of PARP-1 and PARP-2 (Table 1). The Iniparib, originally developed as a non-competitive inhibitor of PARP-1, showed early successes in clinical trials, but it is a non-specific and weak inhibitor of PARP-1 (Patel et al., 2012). Hence this review will focus on the results obtained with competitive PARPi.

**Table 1:** **Different PARPi currently in clinical trials and their relative inhibitory potential against PARP-1 and PARP-2 (adapted from Davar et al., 2012)**.

Inhibitor	Other name(s)	IC_50_/Ki	IC_50_/Ki for PARP-1	IC_50_/Ki for PARP-2	Trial status	Type of cancer(s)
Olaparib	AZD2281 KU0059436	IC50	5 nM	1 nM	Phase I/II singly or combination	Breast, ovarian, colorectal, solid tumors, pancreatic, prostate, carcinoma of esophagus, head and neck squamous cells carcinoma, gastric, NSCLC, brain, CNS, Ewing’s sarcoma, uterine, fallopian tube, etc.
Veliparib	ABT-888	Ki	5.2 nM	2.9 nM	Phase I/II singly or combination	Breast, colorectal, GBM, melanoma, solid tumors, pancreatic, fallopian tube, peritoneal cavity, pancreatic, brain, CNS, lymphoma, multiple myeloma, etc.
Rucaparib	AG014699 PF01367338	Ki	1.4 nM	–	Phase I combined with chemotherapy/phase II singly in BRCA associated status	Breast, ovarian, solid tumors (also diabetes mellitus)
INO-1001	–	IC50	50 nM	–	Phase I/II	Cardiovascular disease/combination with TMZ in melanoma
MK-4827	–	IC50	3.8 nM	2.1 nM	Phase I singly or with chemotherapy/phase II	Ovarian, solid tumors, glioblastoma multiform, melanoma, lymphoma, chronic lymphocytic leukemia, T-cell-pro-lymphocytic leukemia

### PARPi as synthetic lethal monotherapy for DSB repair defective tumors

It was suggested that two mutations should be considered synthetic lethal if cells with either mutation are viable but those with both mutations are non-viable (Dobzhansky, 1946). The first success of this approach was observed in 2005, when two groups showed that PARPi, which is non-toxic to normal cells, is lethal to BRCA1/2 cancer cells that are deficient in HR-mediated repair of DSB (Bryant et al., 2005; Farmer et al., 2005; Helleday et al., 2005). Several clinical trials for different cancers have since been launched with PARPi, and a list of current trials is shown in Table 1.

**Figure 1:**
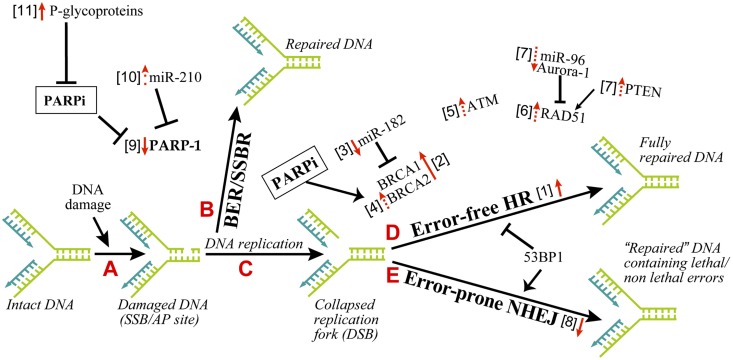
**Mechanisms of resistance to PARPi in cancer therapy**. The principle explanation for the efficacy of PARPi as synthetic lethal therapy in DNA double strand break (DSB) repair defective cancer cells or as a combination therapy with other agents for other cancers rests on the role of PARP-1 in BER and SSBR. As shown in the series of A–E steps, the inefficient repair of DNA single strand breaks by PARP-1-mediated BER in the presence of PARPi would lead to DSB. An inefficient repair of DSB by HR-deficient cancer cells will kill these cells, whereas normal cells with proper DSB repair capacity will survive. The resistance to PARPi can occur in cancer cells by alteration of various parameters, which influence different steps in this pathway. The changes in these factors, i.e., upregulation or downregulation as pointed by the direction of red arrows, is associated with resistance to PARPi. The solid or dashed arrows indicate known or hypothesized mechanisms of resistance to PARPi, respectively. The numbers within square bracket next to the arrows refer to the explanation in the text for this mechanism of PARPi-resistance.

There are different possible mechanisms by which PARPi kill HR-deficient tumor cells (Helleday, 2011). It was initially suggested that constant DNA damage induced by endogenous factors, such as oxidants needs to be repaired by BER in which PARP-1 participates either by binding to SSB or by collaborating with XRCC-1 (Figure 1, steps A and B). Thus, when PARPi block BER, the unrepaired SSB would stall and collapse the replication fork to create DSB (Figure 1, step C). The normal cells would survive by readily repairing these DSB by error-free HR or error-prone NHEJ (Figure 1, steps D or E). However, the DSB would be lethal to HR-deficient BRCA1/2 tumors with an excessive reliance on the error-prone NHEJ repair pathway (Aly and Ganesan, 2011). This scenario is most plausible and is supported by significant evidence, but it does not explain many things, such as lack of accumulation of SSB in PARPi-treated cells or the absence of synthetic lethality by targeting XRCC-1 in BRCA-deficient cells (Helleday, 2011).

Therefore, alternative explanations have been offered for synthetic lethality of PARPi in HR-deficient cells. In brief, it has been shown that PARP-1 binds to and is activated by SSB-intermediates formed during BER, which results in release of PARylated PARP-1 from SSB, which are then repaired (Strom et al., 2011). Thus, it is proposed that in the presence of PARPi, SSB bound to PARP-1 would collapse the replication fork and DSB-mediated lethality will occur in HR-deficient cells (Helleday, 2011). It is also possible that the role of PARP-1 in suppressing the error-prone NHEJ is the target for PARPi-induced lethality in HR-deficient cells, because inhibition or downregulation of multiple components of NHEJ, such as Ku80, Artemis, and DNA-PK, made HR-deficient cells resistant to PARPi (Patel et al., 2011). Finally, it has been suggested that since PARP-1 plays a role in reactivating the stalled replication forks, this step could be a target for PARPi-induced lethality in HR-deficient cells (Helleday, 2011).

Cancer cells are known to carry other conditions that create HR-deficiency or BRCAness, which can make them susceptible to synthetic lethality by PARPi. Three such examples are listed here (Figure 1, step D). (i) The protein kinase ataxia telangiectasia mutated (ATM), a key regulator that senses DNA damage, initiates the protein kinase cascade (Wang and Weaver, 2011) and plays a role in HR, is frequently mutated in lymphoid malignancies. Interestingly, PARPi is synthetic lethal to the ATM mutant lymphoid tumor cells (Weston et al., 2010). (ii) Aurora-1 is frequently amplified and overexpressed in breast cancers (Staff et al., 2010). An overexpression of Aurora-1 induces BRCAness in an otherwise HR-competent PIR12 pancreatic tumor cells by causing an impaired recruitment of key HR-protein RAD51, and sensitizes them to synthetic lethality by PARPi (Sourisseau et al., 2010). (iii) PTEN (phosphatase and tensin homolog), which plays a crucial role in regulating PI3K/Akt-1-mTOR signaling pathway, is frequently mutated or decreased in a wide range of human tumors (Salmena et al., 2008). The PTEN-*null* cancer cells, which are HR-defective due to reduced expression and nuclear localization of RAD51, are sensitive to PARPi (Mendes-Pereira et al., 2009; Dedes et al., 2010; McEllin et al., 2010; Figure 1, step D). Although another study reported that PTEN deficiency in prostate cancer cells is not associated with BRCAness or sensitivity to PARPi (Fraser et al., 2012), suggesting a need for more work in this model.

Finally, PARPi sensitivity has also been reported under circumstances without BRCAness. For example, the depletion of NHEJ components DNA-PK or Ku80 made HR-proficient cells more sensitive to PARPi (Bryant and Helleday, 2006). PARPi sensitivity is also observed in conditions with no apparent defect in any of the DNA repair pathway. The sporadic breast cancer cells overexpressing HER2 (human epidermal growth factor receptor 2) are addicted to overexpression of NF-κB-mediated transcription for survival. Since PARP-1 is a co-activator of NF-κB, the treatment with PARPi abrogates NF-κB-mediated transcription and kills these cancer cells (Nowsheen et al., 2012).

Overall, the ability of PARPi to cause synthetic lethality in cancer cells with BRCAness as well as many other conditions indicates a potential for their use as monotherapy for a wide variety of cancers.

### PARPi in combination therapy for DNA repair proficient tumors

All of the above studies dealing with synthetic lethal effect of PARPi rely on the DNA damage induced by endogenous factors, such as oxidants created during metabolism. Therefore, it is not surprising that PARPi also potentiates lethality of exogenous DNA damaging agents, such as chemotherapeutic agents or ionising radiations (Javle and Curtin, 2011). Such combination therapy has the potential to kill cancer cells with no apparent defect in DNA repair, because chemotherapy induced SSB will be amplified by PARPi to make a large flux of DSB that will overwhelm the normal DSB repair capacity of these tumors and cause death (Figure 1, steps B–E). In the actual clinical conditions for treatment of cancer patients, it is highly likely that PARPi will be used most frequently in combination therapy for DNA repair proficient and even for DNA repair deficient tumors.

## Mechanisms of Resistance to PARPi in Cancer Therapy

There are four categories of known and potential mechanisms of resistance to PARPi in cancer cells, which are described below: (i) increased HR capacity; (ii) altered NHEJ capacity; (iii) decreased levels or activity of PARP-1, and (iv) decreased intracellular availability of PARPi.

### Increased HR capacity

Since pre-existing HR defect is the initial lesion that allows PARPi to kill HR-deficient tumors, any of the following conditions that restore HR could result in the resistance to PARPi (Figure 1, step D, arrow #1).

#### Reverse mutation of *brca*

The resistance of BRCA tumors or cells to PARPi was initially identified to be due to reverse mutations in *brca1/2* and restoration of HR (Figure 1, step D, arrow #2; Ashworth, 2008; Edwards et al., 2008; Sakai et al., 2008; Swisher et al., 2008; Norquist et al., 2011; Barber et al., 2013). For BRCA2, reverse mutation was in part due to intragenic deletion of the c.6174delT mutation and restoration of the open reading frame (Ashworth, 2008). The genomic instability associated with BRCA loss could be a cause for reverse mutations of *brca* (Aly and Ganesan, 2011). Certain BRCA1-deficient tumors carry hypomorphic BRCA1 mutations within its population (Drost et al., 2011); hence a selection of cells with restored BRCA function could confer resistance to PARPi.

#### Overexpression of BRCA via downregulation of miR-182 or PARP-1

BRCA1 expression is negatively regulated by the microRNA miR-182; hence miR-182 overexpression sensitizes BRCA1-proficient breast cancer cells to PARPi, whereas its downregulation made them resistant to PARPi (Moskwa et al., 2011; Figure 1, step D, arrow #3). PARP-1 and its activity is a negative modulator of BRCA2, because PARP-1 binds to the silencer-binding region of the *brca2* promoter (Wang et al., 2008). Hence PARPi mediated suppression of PARP-1 activity could lead to overexpression of BRCA2 and resistance to PARPi (Figure 1, step D, arrow #4).

#### ATM-mediated HR during loss of 53BP1 in BRCA-deficient background

53BP1 is a nuclear protein that plays a key role in DNA repair responses and checkpoint control (Bunting et al., 2010). Together, BRCA1 and 53BP1 determine the balance between NHEJ and HR, because the loss of BRCA1 results in a profound defect in HR and increased NHEJ repair, whereas loss of 53BP1 suppresses NHEJ and promotes HR (Figure 1, steps D–E). While cells with defect in BRCA1 alone were susceptible to PARPi, an additional loss of 53BP1 allowed a partial ATM-dependent HR repair (Aly and Ganesan, 2011), making these cells resistant to PARPi (Cao et al., 2009; Bouwman et al., 2010; Bunting et al., 2010; Brandsma and Gent, 2012; Oplustilova et al., 2012). Thus, increased ATM alone could induce resistance to PARPi (Figure 1, steps D–E, arrow #5).

#### Increased activity of RAD51

RAD51 is a key HR-protein; therefore any factor that increases RAD51 levels or activity can potentially lead to a resistance to PARPi (Figure 1, step D, arrow #6). The levels of RAD51 are suppressed by miR-96 (Wang et al., 2012) and Aurora-1 (Sourisseau et al., 2010) and increased by PTEN (Dedes et al., 2010). Hence, we hypothesize that decreased miR-96 and Aurora-1 or increased PTEN can increase RAD51 and HR activity leading to the resistance to PARPi (Figure 1, step D, arrows #7). This is indirectly supported by the observation that increased RAD51 levels make colon carcinoma cells resistant to the combined treatment of PARPi and temozolomide (Liu et al., 2009).

### Altered NHEJ capacity

One of the causes for synthetic lethality of PARPi in HR-deficient cells is an upregulation of the error-prone NHEJ pathway that is normally suppressed by PARP-1. Hence any decrease in NHEJ capacity in these cells could increase their resistance to PARPi, as shown in BRCA2-deficient cells by inhibition or downregulation of Ku80, Artemis, or DNA-PK (Figure 1, step E, arrow #8; Patel et al., 2011). On the flip side, it has been suggested that normal NHEJ function and the genomic instability mediated by NHEJ could be one of the causes for reversion of the mutation of *brca1/2*, restoration of partial HR capacities and development of resistance to PARPi in HR-deficient tumors (Chiarugi, 2012; Figure 1, step D, arrow #2). Thus, both increased and decreased NHEJ capacity of cells could lead to resistance to PARPi in different contexts.

### Decreased levels or activity of PARP-1

The effectiveness of PARPi in anti-cancer therapy requires that its target PARP-1 is available for inhibition; because in PARPi-treated cells, PARP-1 will still bind to DNA strand breaks but will not be activated to form PAR or facilitate DNA repair events. Hence reduced levels of PARP-1 could result in resistance to PARPi (Figure 1, step B, arrow #9). In fact, PARP-1 levels are significantly decreased in the PARPi and temozolomide-resistant clones of colorectal carcinoma HCT116 cells (Liu et al., 2009). Therefore, it will be interesting to see if alterations in PARP-1 levels during different stages in tumor development are also associated with a corresponding change in sensitivity to PARPi. For example, levels of miR-210, which suppresses PARP-1 expression, are initially decreased when normal breast cells are transformed to ductal carcinoma *in situ*, and they are increased during further transition to the invasive ductal carcinoma stage (Volinia et al., 2012). It will be interesting to test in such a series of samples, whether these changes in miR-210 are inversely associated with alterations in the levels of PARP-1 and directly correlated with the resistance to PARPi (Figure 1, step B, arrow #10). There have been reports of a correlation between the abundance of cytoplasmic PARP-1 and higher sensitivity to chemotherapy in breast cancer samples (Domagala et al., 2011; von Minckwitz et al., 2011; Klauke et al., 2012). However, cytoplasmic PARP-1 was detected at a very low frequency in these tumors, and since we do not know any role for cytoplasmic PARP-1 in DNA damage responses, it is difficult at this moment to rationalize the link between cytoplasmic PARP-1 and resistance to PARPi.

The effectiveness of PARPi is also linked to the catalytic activity of PARP-1. Hence any factor that decreases the activity of PARP-1 could influence the efficacy of PARPi. The cancer cells with normal levels of PARP-1 but decreased enzymatic activity as noted by reduced level of endogenous PARylation are more resistant to PARPi (Oplustilova et al., 2012; Figure 1, step B, arrow #9). As a corollary, HR-deficient tumor cells with higher endogenous PARylation activity are more sensitive to PARPi (Gottipati et al., 2010).

Variant forms of PARP-1 with decreased catalytic activity, such as those created by small nucleotide polymorphism (SNP), could make cancer cells resistant to PARPi. In human cancers, some SNP have indeed been found to some extent, such as V^762^/A (Lockett et al., 2004; Wang et al., 2007; Zaremba et al., 2009) or M^129^/T and E^251^/K (Ogino et al., 2010). However, there is no consensus as to whether V^762^/A reduces enzyme activity and other mutants do not have significant effect on enzyme function. Thus, it is difficult to predict the effect of SNP on the effectiveness of PARPi.

### Decreased intracellular availability of PARPi

A cancer cell that can efficiently throw PARPi out of the cell can become relatively resistant to this therapy. The p-glycoproteins (P-gp) also called multi-drug resistance proteins are involved in the efflux of PARPi (Figure 1, step A, arrow #11), because P-gp inhibitors prevent the decrease of PARPi in HCT116 colon cancer cells (Oplustilova et al., 2012) and re-sensitize PARPi-resistant BRCA-1 deficient cells to PARPi (Rottenberg et al., 2008). In the mouse mammary tumor models, PARPi was more effective when P-gp knockout condition was added to BRCA-1 deficient cells (Jaspers et al., 2012). The P-gp belong to ABC transporter family which is inhibited by ADP-ribose, a product of catalytic activity of PARP-1 (Dumitriu et al., 2004). Therefore, it is feasible that PARPi that would prevent formation of ADP-ribose can permit full activity of P-gp to eliminate PARPi from the cells. Nonetheless, more work is needed in this domain because the resistance to drug via upregulation of P-gp has not yet been shown in human tumoral tissues (Borst, 2012).

## Conclusion

In cancer treatment with PARPi, the personalization of therapy is important because many factors can influence the efficiency of PARPi, such as HR and NHEJ status, PARP-1 levels or its activity and finally other factors that influence intracellular concentrations of PARPi. Therefore, it would be necessary to assess the status of these controlling factors before beginning the treatment with PARPi (Lord and Ashworth, 2012; Ratner et al., 2012). A thorough understanding of different mechanisms for the resistance to PARPi will permit us to design better PARPi monotherapy as well as combination therapy, and will allow us to identify conditions that can re-sensitize tumor cells to PARPi; and thus treat cancer patients more efficiently.

## Conflict of Interest Statement

The authors declare that the research was conducted in the absence of any commercial or financial relationship that could be construed as a potential conflict of interest.
